# Editorial: Antimicrobial Stewardship in Low- and Middle-Income Countries

**DOI:** 10.3389/fpubh.2020.617000

**Published:** 2020-12-02

**Authors:** Inge C. Gyssens, Heiman F. Wertheim

**Affiliations:** ^1^Department of Internal Medicine and Radboud Center for Infectious Diseases, Nijmegen, Netherlands; ^2^Faculty of Medicine and Health Sciences, Hasselt University, Hasselt, Belgium; ^3^Department of Medical Microbiology and Radboud Center for Infectious Diseases, Nijmegen, Netherlands

**Keywords:** antimicrobial resistance (AMR), intervention, antimicrobial stewardship (AMS), low-and middle-income countries, antimicrobial consumption

Antimicrobial stewardship (AMS) is a means to achieve responsible use of antimicrobial drugs. While decreased consumption of antibiotics may be an appropriate target in overconsuming high-income countries (HICs), increased access may be an important goal in low-and middle-income countries (LMICs) as individuals still die from infectious diseases due to insufficient access (1). Surveillance of antimicrobial resistance (AMR) and antimicrobial consumption (AC) are two important pillars of AMS.

National surveillance programs of AMR providing yearly reports and/or interactive data platforms are well-developed in Europe (2) and in other HICs, e.g., Australia (3) and Japan (4). Thailand, an upper-middle-income country, is among the few countries in Southeast Asia with an established AMR surveillance system (5). In 2015 the WHO established the Global Antimicrobial Resistance Surveillance System (GLASS) for collecting official national AMR data in selected bacterial pathogens causing common infections in humans (6). GLASS participation is increasing. However, for AMR surveillance you need good quality data from clinical microbiological laboratories. Often there is limited access to diagnostics in LMICs and if available, the quality can be an issue. Therefore, data are still scarce and in need of quality improvement (7).

Surveillance of AC is less developed as compared to AMR but highly needed. The first GLASS call for data on national AC will be conducted in 2020. However, there are AC data from a number of LMICs in the first global 2018 report (8). In Europe, ECDC issues yearly reports from European Surveillance of Antimicrobial Consumption Network ESAC-net. In 2018, the average total consumption of antibiotics for systemic use in the EU/EEA was 20.1 DDD per 1 000 inhabitants per day with a wide range: 9.7–34.0 (9). Data for comparison in LMICs have been collected. Farooqi et al. studied the 5-years trends (up to 2012) in consumption of major antibiotic classes in India. They found that per capita AC in the retail sector in India has increased by ~22%, but data were still low as compared to ESAC-Net countries (10). Recently, Klein et al., using the same source for 76 countries reported that the AC rate in LMICs has been converging to (and in some countries surpassing) levels typically observed in HICs (11). If part of this AC means increased access, this would be a move in the right direction. However, responsible antibiotic use can only be determined by quality of use evaluation. This requires clinical expertise and access to diagnostics to determine whether an antibiotic was used responsibly. Access to diagnostics is another major issue in LMICs.

While there are many articles on AMS interventions in HICs, the literature on interventions in LMICs is still relatively scarce. In a systematic review and meta-analysis of 77 AMS intervention papers from Asia, only 12 were from MICs (12).

Many reviews have been published about the challenges of AMS in LMICS. These include lack of governmental and political commitment, inadequate funding of healthcare and absence of health insurance, overcrowded healthcare systems, weak regulatory systems, the lack of microbiological expertise and infrastructure, absence of electronic health record systems, limited knowledge of infectious diseases and medicines, substandard medicines and shortage of medicines and trained manpower (7).

In 2019, we launched a call for submissions to the Frontiers' Topic series “Antimicrobial stewardship in LMICs.” We were particularly looking for AMS interventions. Fifteen manuscripts were submitted. Following peer review, eight manuscripts were accepted. All but one focus on AMS in hospitals. Of these eight, only three are original articles describing AMS interventions. Three are qualitative research papers.

Sangeda et al. aimed at assessing hospital AMR surveillance and ASP implementation in hospitals in Tanzania in the year following the launch of the National Action Plan (NAP) for AMR. Using a structured questionnaire, they describe that all 39 (100%) respondents were aware of the presence of AMR in Tanzania, but only 26 (66.7%) were aware of the presence of the NAP. The article shows that e-mailing the questionnaires to the healthcare personnel after a first telephonic contact was feasible at low cost.

Mathew et al. explored the challenges of implementing AMS in Indian hospitals. Interestingly, many of the physicians interviewed cited perceived patient satisfaction as one of the reasons for increased antibiotic use. The determining physician factors included empiric treatment needs, outbreak of diseases, absence of education programmes, and fear of litigation. The promotional activities by companies and antibiotics being a major source of income for small hospitals, affect use patterns. The qualitative information on challenges obtained in this setting shows many similarities with HICs. However, two major sustainability measures, i.e., actions at the governmental level to ensure an antibiotic policy and microbiology labs in all hospitals are implemented in many HICs, and should be prioritized.

Afari-Asiedu et al. report on household surveys in the community in Ghana as part of the international ABACUS project. They found that inappropriate antibiotic use was high and influenced by out-of-pocket payment for healthcare, seeking healthcare outside health centers, pharmacies, and buying antibiotics in installments due to cost. They advocate for the ministry of health and healthcare agencies in Ghana to enhance healthcare access and healthcare insurance, and to provide affordable antibiotics. The study will contribute to the baseline data and surveillance activities of the Ghana National AMR platform.

Regarding interventions on diagnostics, Budayanti et al. report on a microbiological surveillance program of a tertiary referral hospital on Bali, Indonesia. Their data will contribute to the resistance mapping in Indonesian hospitals and provide a frame of reference for developing local guidelines to promote antimicrobial stewardship. Yansouni et al. describe the feasibility of a practical intervention bundle aimed at implementing sustainable clinical bacteriology services at a tertiary hospital in Ethiopia. With external support at the start and long-term supervision they were able to implement a laboratory system with quality control and prepare for future accreditation. This is another important step of many to improve quality microbiology laboratory access.

Regarding antimicrobial use, Gebretekle et al. describe a pharmacist-led laboratory-supported intervention at the same referral hospital. The AMS intervention focused on duration of antibiotic treatment was feasible and had good acceptability. Cessation of audit-feedback activities was associated with immediate and sustained increases in antibiotic consumption, and worse clinical outcomes. This study illustrates that we need to invest in scalable teaching programs for staff to be able to do quality feedback systems in LMICs.

Two articles of the Topic are looking at the future of AS in LMICs. Kakkar et al. propose a model of delivery of AS interventions for various LMIC settings, based on their former experience in a tertiary hospital in India. Interestingly, they also describe a major role for clinical pharmacologists and involved residents of the audited units for their intervention. They state that proven AMS interventions need to be contextualized and tested before they can be successfully employed in LMICs on a wider scale, and propose to rely on the plan-do-check-act (PDCA) model. Tattevin et al. and a multidisciplinary panel advocate for increased international collaborations through the dissemination of high-quality documents and educational material that may be shared, adapted where needed, and adopted worldwide.

Since we launched our call, support by agencies and various organizations has gained momentum. Toolkits for ASP in LMICs are made available by WHO (13), The Center for Disease Dynamics, Economics and Policy (CDDEP) supporting a program called the Global Antimicrobial Resistance Partnership (GARP) (https://cddep.org/projects/global-antibiotic-resistance-partnership/) and the non-government agency ReAct (https://www.reactgroup.org/toolbox)(7).

## Author Contributions

IG and HW conceived and proposed the topic and wrote the editorial. All authors contributed to the article and approved the submitted version.

## Conflict of Interest

The authors declare that the research was conducted in the absence of any commercial or financial relationships that could be construed as a potential conflict of interest.
